# Redox-Dependent Conformational Switching of Diphenylacetylenes

**DOI:** 10.3390/molecules190811316

**Published:** 2014-07-31

**Authors:** Ian M. Jones, Peter C. Knipe, Thoe Michaelos, Sam Thompson, Andrew D. Hamilton

**Affiliations:** 1 Chemistry Research Laboratory, Department of Chemistry, University of Oxford, 12 Mansfield Road, Oxford OX1 3TA, UK; E-Mails: ian.jones@aya.yale.edu (I.M.J.); peter.knipe@chem.ox.ac.uk (P.C.K.); 2 Department of Chemistry, Yale University, P. O. Box 208107, New Haven, CT 06520-8107, USA; E-Mail: thoe.michaelos@yale.edu

**Keywords:** molecular switch, ferrocene, hydrogen bonding, amide bond, redox, translational isomerism

## Abstract

Herein we describe the design and synthesis of a redox-dependent single-molecule switch. Appending a ferrocene unit to a diphenylacetylene scaffold gives a redox-sensitive handle, which undergoes reversible one-electron oxidation, as demonstrated by cyclic voltammetry analysis. ^1^H-NMR spectroscopy of the partially oxidized switch and control compounds suggests that oxidation to the ferrocenium cation induces a change in hydrogen bonding interactions that results in a conformational switch.

## 1. Introduction

Conformational dynamism in the face of changing cellular redox conditions is essential to the survival of living organisms. Protein structure, and thus activity, is often regulated by redox-dependent disulfide bonds [1,2] and metal coordination [3,4]. These natural mechanisms of control inspired us to explore transforming the H-bonded diphenylacetylene (DPA) scaffold into a redox-dependent switch. Much work has been carried out to characterize synthetic systems that can change conformation in response to oxidation state. The most well known of these involve the use of redox active catenanes, [5,6,7,8] metal coordination compounds, [9,10,11,12,13,14,15] rotaxanes, [11,12,14,16,17,18,19,20,21,22] π-systems, [23,24,25,26,27,28] and crowded alkenes [29]. These systems are finding increasing application in solid-state electronic devices. Of particular note are the [2]-rotaxane systems in which a H-bond donating macrocycle shuttles between a strong H-bond accepting succinamide station and a weaker naphthalimide H-bond acceptor [30]. It has been demonstrated that reduction of the naphthalimide group to the radical anion increases its H-bond acceptor strength relative to the succinamide. This change causes the H-bond network to reconfigure, leading to a conformational switch. Analogously it should be possible to design a conformational switch in which a H-bond network reconfigures due to a redox-dependent modulation of a H-bond donor. Previous work has shown that the conformational equilibrium in H-bonded DPA’s can be controlled by increasing the H-bond donation strength of one NH relative to the other [31,32]. This H-bond strength is readily adjusted through the conjugation of electron-donating or -withdrawing groups to the amide NH.

**Figure 1:**
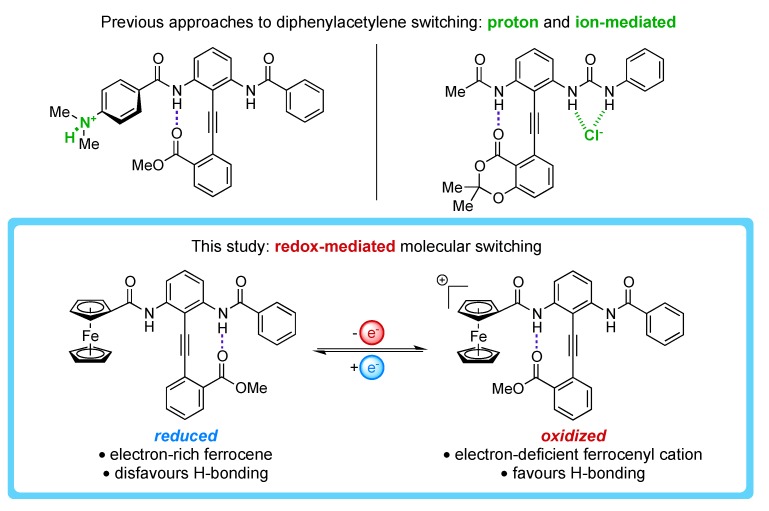
The conceptual transformation from ion-mediated switches to a redox-dependent analogue.

By this same principle, conjugating a ferrocene (Fc) to the H-bond network should mediate H-bond donor strength in a redox-dependent fashion (Figure 1). Although used extensively in the field of sensors, [33,34,35] ferrocene has received little attention as an actuator of redox-dependent conformational switching [36]. Fc is slightly electron- donating [37,38] as compared to a phenyl group, which suggests that the H-bonded equilibrium will be biased away from the ferrocenyl amide (FcA) in the neutral state (“reduced”, Figure 1). However, ferrocene is also known to undergo a reversible single electron oxidation to the ferrocenium cation [39]. Studies on 1-ferrocenylcarboxamide systems show that the oxidation of Fc withdraws electron density from the amide NH bond, creating a stronger H-bond donor [40,41,42,43,44,45,46]. Thus, it is hypothesized that oxidation to Fe(iii) should induce a switch in the H-bonded equilibrium toward the FcA (“oxidized”, Figure 1).

## 2. Results and Discussion

### 2.1. Synthesis

**Scheme 1:**
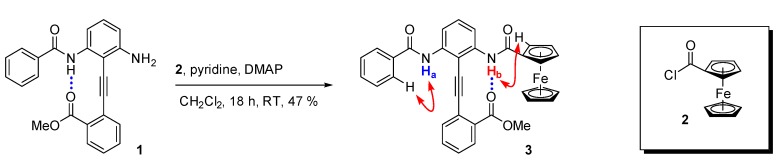
Synthesis of a benzamido/FcA-substituted diphenylacetylene molecular switch (the red arrows represent NOE correlations).

In order to test this hypothesis we synthesized diphenylacetylene **3** via an amide bond formation between aniline **1** [31] and ferrocenoyl chloride **2** (Scheme 1).

### 2.2. Solid-State Analysis of Conformation

**Figure 2:**
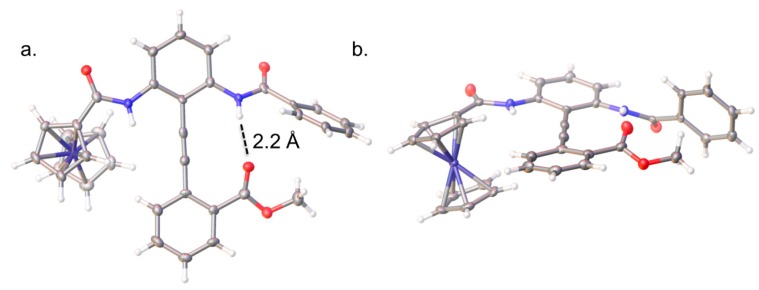
The single crystal X-ray structure of **3** viewed (**a**) perpendicular to the phenyl-alkyne plane; and (**b**) down the phenyl-alkyne axis. The ORTEP ellipsoids are shown at the 50% probability level; white = H, grey = C, red = O, blue = N, purple = Fe.

Insight concerning the conformation of diphenylacetylene **3** was obtained from single crystal X-ray diffraction following recrystallization from 1:1 methanol/chloroform (Figure 2, see also Section 3.4.). The H-bond acceptor is bound to the benzamide NH with an N•••OC distance of 3.1 Å. The steric clash between the methyl ester and the phenyl ring causes the ring to rotate 35° out of the amide plane.

The solid-state data confirm the predicted conformation, which is presumably favoured due to the electron-donating character of the Fc group reducing the H-bond donor capability of the corresponding amide, as well as the steric demands of the large sandwich complex.

### 2.3. Determining the Solution Phase Conformational Bias

**Scheme 2:**
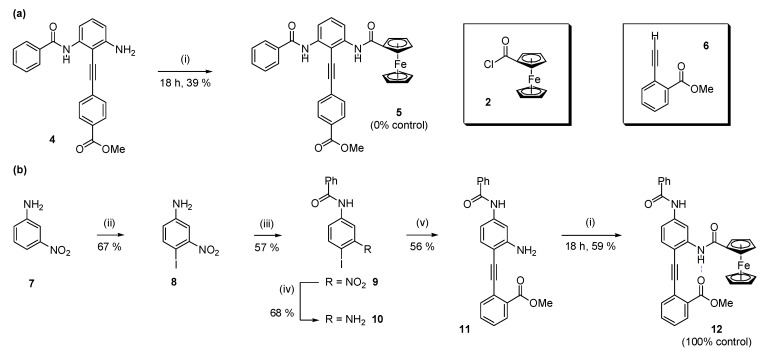
Synthesis of benzamido/FcA-substituted diphenylacetylene compounds: (**a**) 0% control **5**; and (**b**) 100% control **12**.

As for the anion [47] and pH-dependent [32] switches described previously, the conformational bias can be determined by comparison of the ^1^H-NMR spectrum of **3** with a set of control compounds. *para*-Substituted benzoic ester **5**, which is incapable of intramolecular H-bonding, was synthesized via amide coupling of aniline **4** [31] with ferrocenoyl chloride (Scheme 2). The second control molecule, **12**, was prepared from 3-nitroaniline via an adaptation of a literature route: [31] iodination of 3-nitroaniline, [48] followed by amidation, nitro-group reduction, Sonogashira coupling and coupling with ferrocenoyl chloride, afforded the desired bis(amide).

*Regent and conditions*: (i) Pyridine, DMAP, CH_2_Cl_2_, **2**; (ii) I_2_, Ag_2_SO_4_, MeOH, 3 h; (iii) PhCOCl, pyridine, DMAP, CH_2_Cl_2_, 5 h; (iv) SnCl_2_**^.^**2H_2_O, EtOAc, 18 h; (v) PdCl_2_(PPh_3_)_2_ (6 mol%), CuI (10 mol%), **6** (1.2 eq.), DMF/NEt_3_ (1:1 *v*/*v*), 70 °C, 3 h. All reactions were run at room temperature unless specified otherwise; Ac = acetyl, DMAP = 4-dimethylaminopyridine, DMF = *N*,*N*-dimethylformamide, Me = methyl, Et = ethyl, Ph = C_6_H_5_.

**Figure 3:**
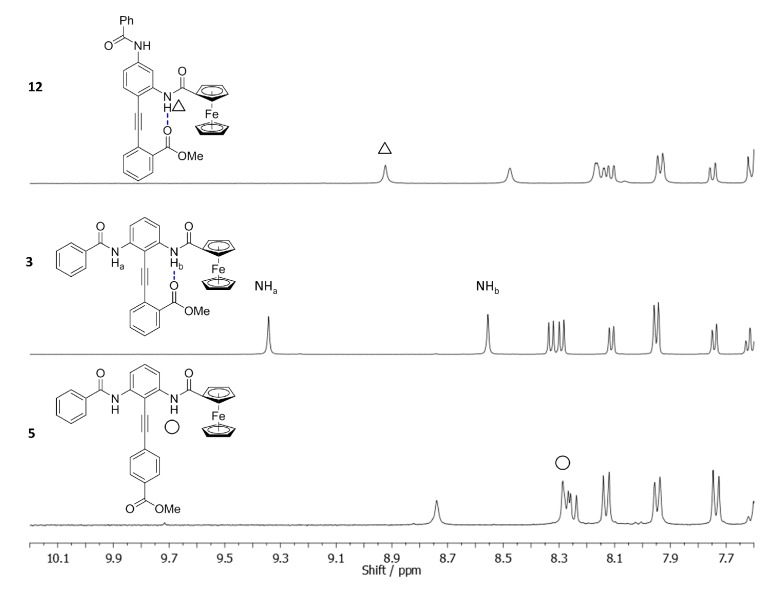
The ^1^H-NMR conformational analysis for **3** using the 100% **12** and 0% **5** controls (4.0 mM, CDCl_3_, peak assignments determined by NOE and COSY experiments).

The first of these, **5**, estimates the chemical shift of the FcA NH in the absence of H-bonding by positioning the H-bond acceptor *para* to the alkyne linkage (0% control). The second control, **12**, estimates the chemical shift of the FcA NH when it is completely H-bonded (100% control). The spectra of these controls are compared with parent compound **3** in Figure 3. 

Using Equation (1), the conformational bias is calculated to be 14.3%, which equates to a ratio of ~1.4:1 toward the benzamide NH, in agreement with the preference demonstrated in the solid state (*vide supra*):

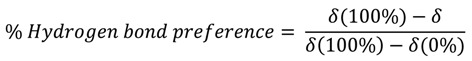
(1)


### 2.4. Characterizing the Redox Properties of Switch **3** with Cyclic Voltammetry

**Figure 4:**
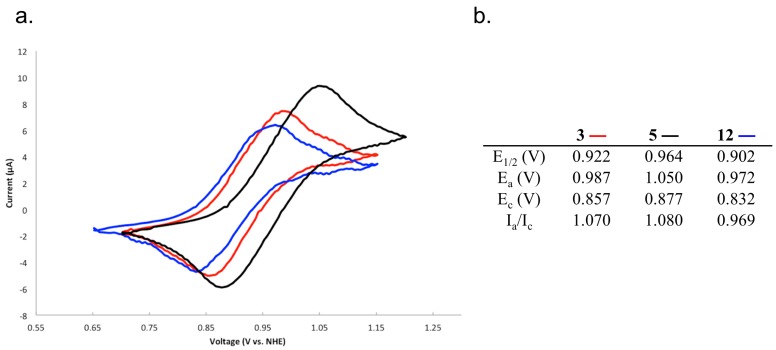
(**a**) Cyclic voltammograms (CV) for **3** (red), **5** (black), and **12** (blue); compounds at 2.0 mM with 0.1 M NBu_4_PF_6_ in dichloromethane with a scan rate of 100 mV∙s^−1^. Positive currents correspond to oxidation. Potentials were measured relative to the ferrocenium/ferrocene redox couple as an internal standard, and are reported relative to the NHE; (**b**) Derived electrochemical parameters (see Experimental Section 3.1. for further details).

The redox properties of the ferrocene-containing compounds **3**, **5** and **12** were examined by cyclic voltammetry (Figure 4, Figure 4b) All three compounds exhibit one-electron oxidation waves characteristic of the Fe(ii)/Fe(iii) couple. The peak potential separations (ΔE_p_ = 130–170 mV) are greater than the 59 mV expected for a reversible one-electron process; however, these numbers are on the order of the ferrocene internal reference (ΔE_p, Fc_ = 165 mV at 100 mV∙s^−1^), and the ratio of peak currents (I_a_/I_c_) are all close to unity, indicating that these are chemically reversible processes.

The oxidation potentials (E_1/2_) have the order **5** > **3** > **12**, consistent with increasing H-bonding to ferrocenylcarboxamides causing a depression in the ferrocene E_1/2_ [40,41,45,46].

### 2.5. Characterizing the Redox Properties of Switch **3** by Chemical Oxidation

Having shown that putative switch **3** undergoes reversible oxidation, we attempted to use the ^1^H-NMR conformational assay described above to analyze [**3**]^+^. The formation of paramagnetic Fe(iii) caused broadening of resonances, rendering ^1^H-NMR spectral interpretation difficult. However, Heinz* et al.* have recently examined the NMR spectra of partially oxidized Fc compounds [36,49]. They report that, while partial oxidation causes the Fc signals to broaden and shift downfield, other resonances belonging to protons further from the FcA group remain sharp.

**Figure 5:**
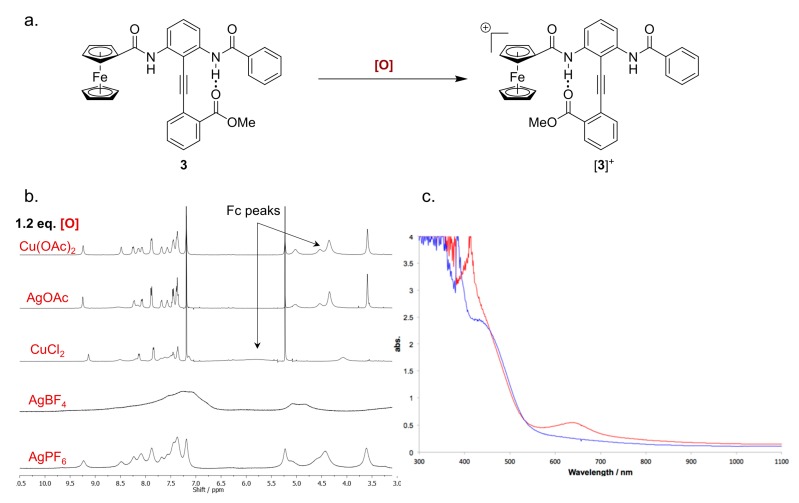
(**a**) Chemical oxidation of **3** to [**3**]^+^; (**b**) An array of ^1^H-NMR spectra acquired after treatment of **3** with various chemical oxidants for 2 h (4.0 mM in CDCl_3_, peak assignments determined by NOE and COSY experiments); (**c**) The UV-vis absorbance spectra of **3** before (blue) and [**3**]^+^ after (red) oxidation with CuCl_2_ (4.0 mM, CH_2_Cl_2_, see the Experimental Section for further details).

In order to determine the conformational ratio of a partially oxidized **3**, conditions must be identified that bring about the partial oxidation of this Fc DPA system. Figure 5a shows the NMR spectra of **3** after exposure to various chemical oxidants. The extent of oxidation was qualitatively assessed by examining the cyclopentadienyl peaks, 5.1–4.0 ppm, which are known to broaden and shift downfield upon oxidation. After two hours only copper(ii) chloride and silver tetrafluoroborate caused the expected change in the signals of **3**. Additionally, copper(ii) chloride caused some broadening of other signals in the aromatic region but the NH resonances are both identifiable. It is plausible this broadening is due to one or more of the oxidation states of copper acting as a Lewis acid with acceptor lone pairs of the switch compound. Silver tetrafluoroborate caused the loss of all spectral definition, most likely due to more complete oxidation (Figure 5b). The presence of a ferrocenium group was also confirmed by the appearance of the characteristic peak at 640 nm in the absorbance spectrum (Figure 5c) [50].

### 2.6. Paramagnetic NMR Characterization of the Solution Phase Conformation

**Figure 6:**
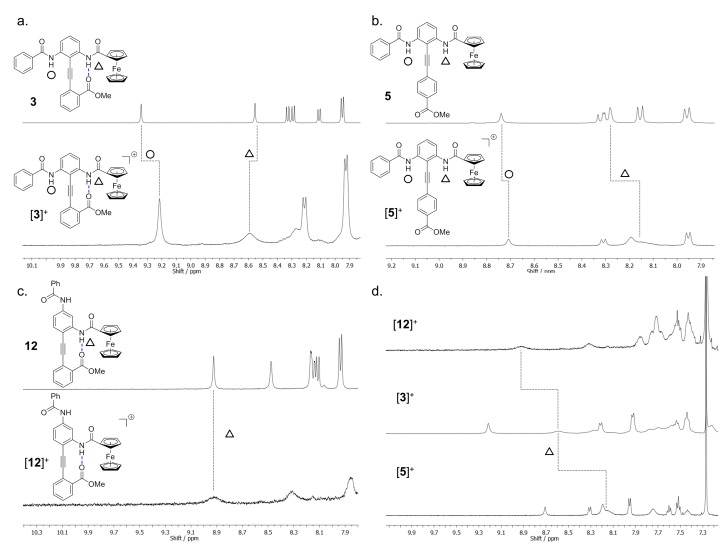
A comparison of the ^1^H-NMR spectra of (**a**) **3** and [**3**]^+^; (**b**) **5** and [**5**]^+^; (**c**) **12** and [**12**]^+^; and (**d**) the conformational analysis of [**3**]^+^ (peak assignments determined by NOE and COSY experiments).

With a procedure to effect the partial oxidation of **3** in hand, we next began to investigate the influence of oxidation on the spectral features. Figure 6a compares the downfield NMR spectrum of [**3**]^+^ with that of neutral **3**. The FcA NH is broadened, and shifted downfield by 0.05 ppm while the benzamide NH migrates 0.14 ppm upfield. The singlet corresponding to the methyl ester was also affected, broadening and shifting downfield by ~0.6 ppm.

To understand the meaning of these changes, control compound **5** was used to examine the effect of oxidation in the limiting case of 0% H-bond interaction. This compound was treated with copper(ii) chloride for two hours and, after the presence of the ferrocenium was confirmed by UV-vis, its ^1^H-NMR spectrum was acquired. This spectrum indicated that FcA NH and benzamide NH signals of [**5**]^+^ shift *upfield* by 0.11 and 0.03 ppm respectively, relative to **5** (Figure 6b). The methyl ester resonance was unaffected by oxidation, presumably due to its isolation *para* to the alkyne. Lastly, **12** was oxidized with copper(ii) chloride; the partially oxidized spectra of **12** and [**12**]^+^ are shown in Figure 6c. In this case the FcA NH broadens but is not shifted, while the benzamide NH is not identifiable. Furthermore, the methyl ester peak was broadened to such an extent that it was not observable in the partially oxidized spectrum. Attempts to monitor IR stretching frequencies of the carbonyl, or NH, regions to provide conformational data was not possible due to poor resolution of overlapping signals.

Taken together, these observations suggest that the H-bonded equilibrium is changing as the Fc group becomes oxidized. The benzamide NH resonance, for example, shifts upfield both in the absence and presence of the intramolecular H-bond (Figure 6a,b), but the signal migrates further when the H-bond is engaged ([**3**]^+^). This increased upfield shift can be explained by a switch of the H-bond acceptor away from the benzamide NH, which causes this proton to be more shielded. The Fc^+^ amide NH shifts upfield in the absence of H-bonding upon oxidation (Figure 6b), but shifts downfield when the H-bonded equilibrium is engaged (Figure 6a). A switch of the H-bond acceptor towards the Fc^+^ amide NH, causing this proton to become deshielded, would explain the downfield shift in the spectrum of [**3**]^+^. To estimate the effect of partial oxidation on the conformational equilibrium, the spectra of [**3**]^+^, [**5**]^+^ and [**12**]^+^ are directly compared (Figure 6d) using the same method as in the neutral case. This comparison suggests that the equilibrium is biased toward the Fc^+^ amide NH by 15.6%, or ~1.4:1 in favour of the Fc^+^ amide (compared with 1.4:1 in favour of the benzamide prior to oxidation) under these partially oxidized conditions. Since the ^1^H-NMR resonances corresponding to the parent unoxidized species are not evident it is probable that there is fast exchange between the unoxidized and oxidized forms, leading to average peak positions. Whilst this makes precise quantification of the bias more difficult to establish, the overall trends hold thus providing a semi-quantitative measurement.

## 3. Experimental Section

### 3.1. General Methods

Dichloromethane, tetrahydrofuran, and *N*,*N*-dimethylformamide were dried using an Innovative Technology SPS-400 dry solvent system. Anhydrous methanol, ethanol, isopropanol, and dimethyl sulfoxide were purchased from Sigma-Aldrich and used directly from their SureSeal^TM^ bottles. All reactions were performed under an atmosphere of dry nitrogen in oven- or flame-dried glassware and were monitored by thin-layer chromatography (TLC) using silica gel (visualized by UV light). Aqueous solutions were saturated unless otherwise stated. ^1^H and ^13^C NMR spectra were recorded on 400 or 500 MHz Bruker or 500 MHz Varian instruments. Chemical shifts (δ) are reported in parts per million after reference to residual isotopic solvent. Spectra measured in CDCl_3_ were referenced to 7.27 and 77.16 ppm for ^1^H and ^13^C. Spectra measured in CD_2_Cl_2_ were calibrated to 5.32 (^1^H) and 53.52 (^13^C) ppm. Coupling constants (*J*) are reported in Hertz (Hz). Proton assignments were pre-formed using MestReNova “multiplet reporter script”, and were edited by hand. High-resolution mass spectra were measured on a 9.4 T Bruker Qe FT-ICR MS and values are the average of three measurements. All chemical drying was pre-formed with sodium sulfate unless otherwise stated. Cyclic voltammetry was performed with an EG&G Princeton Applied Research Model 273A potentiostat/galvanostat using platinum disc (1.6 mm diameter) working electrode, a platinum counter electrode, and a silver wire pseudo-reference electrode in a conventional three-electrode cell. Anhydrous dichloromethane was used as the solvent. The supporting electrolyte was 0.10 M tetrabutylammonium hexafluorophosphate, and bubbling with nitrogen deoxygenated the solution. Polishing with alumina slurry, followed by solvent rinses, cleaned the platinum disc working-electrode. The concentration of the electroactive compound was 2.0 mM. The potential of the pseudo-reference electrode was determined using the ferrocenium/ferrocene redox couple as an internal standard (with E_1/2_ taken as 0.690 V* vs.* NHE in dichloromethane). For aqueous solutions, the supporting electrolyte was 0.1 M sodium sulfate. All reported voltammograms were recorded at a 100 mV∙s^−^^1^ scan rate. All potentials listed in this manuscript are referenced to the normal hydrogen electrode (NHE) unless otherwise stated.

### 3.2. Synthetic Procedures

*Methyl 2-((2-amino-6-benzamidophenyl)ethynyl)benzoate* (**1**). Methyl 4-((2-amino-6-benzamidophenyl)ethynyl)benzoate (**4**) and methyl 2-ethynylbenzoate (**6**) were prepared according to a literature procedure [31].

*Ferrocenoyl chloride* (**2**). Oxalyl chloride (0.058 mL, 0.66 mmol) was added dropwise over 1 min to a solution of ferrocene monocarboxylic acid (0.0777 g, 0.338 mmol) in dichloromethane (3.0 mL) and *N*,*N*-dimethylformamide (1–3 drops). The mixture stirred for 30 min and concentrated with a stream of nitrogen gas, and then subjected to vacuum for 10 min. The resultant crude oil was used without further purification.

*Methyl 2-((2-benzamido-6-(ferrocenecarboxamido)phenyl)ethynyl)benzoate* (**3**). Pyridine (0.011 mL, 0.14 mmol) was added dropwise to a solution of amine **1** (0.0500 g, 0.135 mmol) and 4-dimethylaminopyridine (ca. 1 mg) in dichloromethane (4.5 mL). A solution of ferrocenoyl chloride **2** (0.9 mL, 0.3 M, 0.27 mmol) was added dropwise over 3 min and the mixture stirred for 18 h. Following dilution with dichloromethane and washing with 2 *N* hydrochloric acid, sodium hydrogen carbonate, and brine, the organic layers were dried over magnesium sulfate and concentrated *in vacuo*. The residue was purified by column chromatography on silica gel (100% chloroform to 9:1 chloroform/ethyl acetate) to give the title compound **3** (0.037 g, 47%) as an orange solid; R_f_ 0.5 (9:1 chloroform/ethyl acetate); ^1^H-NMR (500 MHz, CDCl_3_): δ_H_ 9.34 (s, 1H), 8.56 (s, 1H), 8.33 (d, *J* 8.3, 1H), 8.29 (d, *J* 8.3, 1H), 8.11 (d, *J* 7.8, 1H), 7.95 (d, *J* 7.4, 2H), 7.74 (d, *J* 7.6, 1H), 7.61 (t, *J* 7.5, 1H), 7.51 (m, 2H), 7.44 (m, 3H), 4.92 (s, 2H), 4.43 (s, 2H), 4.21 (s, 5H), 3.57 (s, 3H); ^13^C-NMR (125 MHz, CDCl_3_): δ_C_ 169.0, 166.6, 165.5, 140.5, 139.9, 135.5, 133.2, 132.6, 131.8, 131.2, 131.0, 130.5, 129.0, 128.5, 127.9, 123.1, 115.0, 114.9, 102.5, 101.8, 86.1, 77.4, 76.1, 71.1, 70.1, 68.6, 52.3; HRMS: found 583.1315; C_34_H_27_FeN_2_O_4_^+^ [M+H]^+^ requires 583.1242.

*Methyl 4-((2-benzamido-6-(ferrocenecarboxamido)phenyl)ethynyl)benzoate* (**5**). Pyridine (0.011 mL, 0.14 mmol) was added dropwise to a solution of amine **4** (0.0500 g, 0.135 mmol) and 4-dimethylaminopyridine (ca. 1 mg) in dichloromethane (4.5 mL). A solution of ferrocenoyl chloride **2** (0.9 mL, 0.3 M, 0.27 mmol) was added dropwise over 3 min and the mixture stirred for 18 h. Following dilution with dichloromethane and washing with 2 *N* hydrochloric acid, sodium hydrogen carbonate, and brine, the organic layers were dried over magnesium sulfate and concentrated *in vacuo*. The residue was purified by column chromatography on silica gel (9:1 chloroform/ethyl acetate) to give the title compound **5** (0.0310 g, 39%) as an orange solid; R_f_ 0.41 (9:1 chloroform/ethyl acetate); ^1^H-NMR (400 MHz, CDCl_3_): δ_H_ 8.74 (s, 1H), 8.32 (d, *J* 8.4, 1H), 8.30 (s, 1H), 8.28 (s, 1H), 8.16 (d, *J* 8.2, 2H), 7.96 (d, *J* 7.5, 2H), 7.70 (d, *J* 8.2, 2H), 7.60 (t, *J* 7.3, 1H), 7.51 (m, 2H), 7.47 (d, *J* 8.4, 1H), 4.81 (s, 2H), 4.47 (s, 2H), 4.23 (s, 5H), 3.98 (s, 3H); ^13^C-NMR (125 MHz, CDCl_3_): δ_C_ 168.8, 166.2, 165.2, 139.8, 139.6, 134.9, 132.4, 131.6, 131.3, 131.1, 130.3, 129.1, 127.1, 126.1, 115.0, 114.7, 103.0, 101.0, 83.4, 76.0, 71.4, 70.2, 68.4, 52.6; HRMS: found 583.1270; C_34_H_27_FeN_2_O_4_^+^ [M+H]^+^ requires 583.1242.

*4-Iodo-3-nitroaniline* (**8**). Based on literature procedures [48,51] 3-nitroaniline **7** (0.100 g, 0.72 mmol) was added to a solution of iodine (0.183 g, 0.724 mmol) and silver(i) sulfate (0.223 g, 0.724 mmol) in methanol (15 mL). After 3 h the solution was filtered and the solid re-dissolved in aqueous sodium hydroxide (5%, 7.0 mL). The mixture was heated to boiling and allowed to cool. The precipitate was filtered, collected and purified by column chromatography on silica gel (1:1 hexanes/dichloromethane) to give the title compound **8** (0.128 g, 67%) as a gold solid; R_f_ 0.3 (1:1 hexanes/dichloromethane); ^1^H-NMR (400 MHz, CDCl_3_): δ_H_ 7.70 (d, *J* 8.5, 2H), 7.59 (dd, *J* 2.5, 8.5, 1H), 7.50 (t, *J* 2.2, 2H), 7.29 (d, *J* 2.5, 1H), 7.19 (d, *J* 2.7, 2H), 6.97 (m, 1H), 6.61 (dd, *J* 2.7, 8.5, 2H), 4.07 (s, 5H); HRMS: found 264.9452; C_6_H_6_IN_2_O_2_^+^ [M+H]^+^ requires 264.9396.

*N-(4-Iodo-3-nitrophenyl)benzamide* (**9**). Benzoyl chloride in dichloromethane (1.5 mL, 0.1 M, 0.15 mmol) was added dropwise over 1 min to a solution of amine **8** (0.0337 g, 0.128 mmol) and 4-dimethylaminopyridine (ca. 1 mg) in dichloromethane (1.3 mL). The reaction was stirred for 5 h before dilution with dichloromethane. After washing with 1 *N* hydrochloric acid and brine the solution was dried over magnesium sulfate and concentrated *in vacuo*. The residue was purified by column chromatography on silica gel (1:1 hexanes/dichloromethane) to give the title compound **9** (0.018 g, 57%) as a yellow solid; R_f_ 0.32 (1:1 hexanes/dichloromethane); ^1^H-NMR (400 MHz, CDCl_3_): δ_H_ 8.29 (d, *J* 2.5, 1H), 8.00 (d, *J* 8.6, 2H), 7.88 (dd, *J* 1.3, 8.3, 2H), 7.67 (m, 1H), 7.61 (m, 1H), 7.53 (t, *J* 7.5, 2H); ^13^C-NMR (75 MHz, (CD_3_)_2_SO): δ_C_ 166.0, 152.9, 141.3, 140.2, 134.0, 132.1, 128.5, 127.8, 125.0, 116.2, 79.9; HRMS: found 368.9776; C_13_H_10_IN_2_O_3_^+^ [M+H]^+^ requires 368.9658.

*N-(3-Amino-4-iodophenyl)benzamide* (**10**). Tin(ii) chloride dihydrate (0.153 g, 0.68 mmol) was added to a solution of nitro aromatic **9** (0.050 g, 0.136 mmol) in ethyl acetate (5 mL). The mixture was stirred for 18 h and diluted with ethyl acetate and poured into sodium hydrogen carbonate. The mixture was filtered over Celite^®^ and the filtrate was extracted using ethyl acetate, dried over magnesium sulfate, and concentrated *in vacuo*. The residue was purified by column chromatography on silica gel (2:1 hexanes/ethyl acetate) to give the title compound **10** (0.031 g, 68%) as a white solid; R_f_ 0.5 (2:1 hexanes/ethyl acetate); ^1^H-NMR (400 MHz, CDCl_3_): δ_H_ 7.76 (d, *J* 7.0, 4H), 7.70 (s, 2H), 7.48 (m, 3H), 7.40 (m, 5H), 6.48 (dd, *J* 2.4, 8.5, 2H), 4.07 (s, 1H); ^13^C-NMR (125 MHz, (CD_3_)_2_SO): δ_C_ 165.4, 148.5, 140.2, 138.1, 135.0, 131.5, 128.3, 127.6, 111.1, 106.1, 76.5; HRMS: found 338.9881; C_13_H_12_IN_2_O^+^ [M+H]^+^ requires 338.9916.

*Methyl 2-((2-amino-4-benzamidophenyl)ethynyl)benzoate* (**11**). A solution of **10** (0.031 g, 0.0917 mmol) and methyl 2-alkynylbenzoate **6** (0.0176 g, 0.110 mmol) in *N*,*N*-dimethylformamide (1.0 mL) and triethylamine (1.0 mL) was degassed by nitrogen stream for 10 min. Bis(triphenylphosphine)palladium(ii) dichloride (0.0038 g, 0.0055 mmol) and copper(i) iodide (0.0017 g, 0.0092 mmol) were added and the mixture was heated to 70 °C. The reaction was allowed to stir for 2 h then diluted with ethyl acetate and washed with water. The organic layer was dried and concentrated *in vacuo*. The product was purified by column chromatography on silica gel (2:1 hexanes/ethyl acetate) to give the title compound **11** (0.019 g, 56%) as a yellow solid; ^1^H-NMR (500 MHz, CDCl_3_): δ_H_ 8.03 (d, *J* 7.9, 1H), 7.85 (d, *J* 7.1, 2H), 7.65 (d, *J* 7.7, 1H), 7.55 (t, *J* 7.4, 1H), 7.52 (d, *J* 8.0, 1H), 7.48 (t, *J* 7.6, 2H), 7.42 (s, 1H), 7.34 (m, 2H), 6.74 (d, *J* 8.3, 2H), 5.17 (s, 2H), 3.94 (s, 3H); ^13^C-NMR (125 MHz, CDCl_3_): δ_C_ 166.4, 165.8, 150.9, 139.9, 135.1, 133.6, 132.8, 132.2, 132.0, 130.7, 128.9, 127.3, 127.1, 124.9, 108.8, 104.9, 103.4, 93.7, 92.8, 52.4; HRMS: found 371.1262; C_23_H_19_N_2_O_3_^+^ [M+H]^+^ requires 371.1317.

*Methyl 2-((4-benzamido-2-(ferrocenecarboxamido)phenyl)ethynyl)benzoate* (**12**). A solution of ferrocenoyl chloride **2** in dichloromethane (2.1 mL, 0.065 M, 0.137 mmol) was added dropwise over 10 min to a solution of amine **11** (0.025 g, 0.068 mmol) and 4-dimethylaminopyridine (ca. 1 mg) in dichloromethane (2.6 mL) and pyridine (0.006 mL, 0.07 mmol). The mixture was stirred for 18 h then diluted with dichloromethane. The organic layer was washed with 2 *N* hydrochloric acid, sodium hydrogen carbonate, and brine. After drying and concentration *in vacuo* the residue was purified by column chromatography on silica gel (9:1 chloroform/ethyl acetate) to give the title compound **12** (0.023 g, 59%) as an orange solid; ^1^H-NMR (500 MHz, CDCl_3_): δ_H_ 8.85 (s, 1H), 8.53 (m, 1H), 8.44 (s, 1H), 8.16 (m, 1H), 8.10 (d, *J* 7.9, 1H), 7.90 (d, *J* 8.0, 2H), 7.74 (d, *J* 7.7, 1H), 7.62 (d, *J* 8.9, 1H), 7.58 (d, *J* 7.6, 1H), 7.53 (m, 1H), 7.49 (d, *J* 7.6, 2H), 7.45 (d, *J* 7.7, 1H), 4.99 (s, 2H), 4.37 (s, 2H), 4.16 (s, 5H), 3.89 (s, 3H); ^13^C-NMR (125 MHz, CDCl_3_): δ_C_ 170.1, 166.2, 165.9, 140.3, 139.9, 134.8, 134.1, 133.5, 132.5, 132.1, 130.8, 128.9, 128.4, 127.3, 124.1, 115.1, 111.0, 107.9, 94.6, 90.5, 71.0, 70.1, 69.1, 52.6; HRMS: found 583.1376; C_34_H_27_FeN_2_O_4_^+^ [M+H]^+^ requires 583.1242.

### 3.3. Chemical Oxidation Procedure

Copper(ii) chloride (0.0025 g, 0.019 mmol) was added to a solution of ferrocenyl compound (**3**, **5**, or **12**) in dichloromethane (3.87 mL, 4.0 mM, 0.016 mmol), and the mixture stirred for 2 h, giving a dark greenish solution. The solution was filtered, transferred to a cuvette, and the UV-vis absorbance spectrum obtained to determine the presence of a ferrocenium group. The solution was concentrated and re-dissolved in 3.87 mL of CDCl_3_ for NMR characterization.

### 3.4. Single Crystal X-ray Diffraction

Crystallographic data (excluding structure factors) have been deposited with the Cambridge Crystallographic Data Centre (CCDC: 871793) and copies of these data can be obtained free of charge via the web [52].

#### 3.4.1. Data Collection

An orange prism crystal of FeC_34_H_26_N_2_O_4_ having approximate dimensions of 0.40 × 0.40 × 0.32 mm was mounted in a loop. All measurements were made on a Rigaku R-AXIS RAPID imaging plate diffractometer using filtered Cu-Kα radiation. The crystal-to-detector distance was 127.40 mm. Cell constants and an orientation matrix for data collection corresponded to a primitive monoclinic cell with dimensions: a = 10.3095(3) Å; b = 13.5730(4) Å; β = 97.929(7)°; c = 18.9524(13) Å; V = 2626.7(2) Å^3^. For Z = 4 and F.W. = 582.44, the calculated density is 1.473 g∙cm^−3^. The reflection conditions of: h0l: l = 2n; 0k0: k = 2n, uniquely determine the space group to be: P2_1_/c (#14). The data were collected at a temperature of −180 ± 1 °C to a maximum 2θ value of 130.1°. A total of 105 oscillation images were collected. A sweep of data was done using ω scans from 20.0 to 200.0° in 5.0° step, at χ = 54.0° and ϕ = 180.0°. The exposure rate was 12.0 [s/°]. A second sweep was performed using ω scans from 22.0 to 197.0° in 5.0° step, at χ = 54.0° and ϕ = 270.0°. The exposure rate was 12.0 [s/°]. Another sweep was performed using ω scans from 42.0 to 132.0° in 5.0° step, at χ = 54.0° and ϕ = 0.0°. The exposure rate was 12.0 [s/°]. Another sweep was performed using ω scans from 37.0 to 117.0° in 5.0° step, at χ = 54.0° and ϕ = 90.0°. The exposure rate was 12.0 [s/°]. The crystal-to-detector distance was 127.40 mm. Readout was performed in the 0.100 mm pixel mode.

#### 3.4.2. Data Reduction

Of the 17,074 reflections that were collected, 4,399 were unique (R_int_ = 0.0563). The linear absorption coefficient, μ, for Cu-Kα radiation is 49.717 cm^−1^. An empirical absorption correction was applied which resulted in transmission factors ranging from 0.140 to 0.204. The data were corrected for Lorentz and polarization effects.

#### 3.4.3. Structure Solution and Refinement

The structure was solved by direct methods [53] and expanded using Fourier techniques. The non-hydrogen atoms were refined anisotropically. Hydrogen atoms were refined using the riding model. The final cycle of full-matrix least-squares refinement on F^2^ was based on 4383 observed reflections and 371 variable parameters and converged (largest parameter shift was 0.00 times its esd) with unweighted and weighted agreement factors of:

R1 = ∑ ||Fo| − |Fc||/∑ |Fo| = 0.0504
(2)

wR2 = [∑ (w (Fo^2^ – Fc^2^)^2^)/∑ w(Fo^2^)^2^]^1/2^ = 0.1294 (3)

The standard deviation of an observation of unit weight was 1.10. Unit weights were used. The maximum and minimum peaks on the final difference Fourier map corresponded to 0.58 and −0.86 e^−^/Å^3^, respectively. Neutral atom scattering factors were taken from Cromer and Waber [54]. Anomalous dispersion effects were included in Fcalc; [55] the values for ∆f’ and ∆f” were those of Creagh and McAuley [56]. The values for the mass attenuation coefficients are those of Creagh and Hubbell [57]. All calculations were performed using the CrystalStructure [58] crystallographic software package except for refinement, which was performed using SHELXL-97 [59].

## 4. Conclusions

The importance of redox-dependent conformational switching in Nature inspired the development of an analogous synthetic molecular switch. Previous studies have demonstrated redox switching by modulation of H-bond acceptor strength, but there are few examples in which this is mediated by tuning of H-bond donor ability. Conjugation of a ferrocenylcarboxamide to a diphenylacetylene scaffold provided a model system for redox-dependent conformational switching. X-ray crystallography established that the electron-donating character of the Fc group causes hydrogen bonding to the benzamide to be preferred in the solid state, and ^1^H-NMR analysis confirmed this behavior in solution. Upon partial oxidation the compound undergoes a switching of conformational ratio from 1.4:1 in favour of the benzamide to 1.4:1 in favour of the ferrocenyl amide; however, due to paramagnetism of the Fe(iii) species, ^1^H-NMR analysis of the fully oxidized species was not possible. Cyclic voltammetry also supports the predicted conformational change upon oxidation, by showing that the Fe(ii)/Fe(iii) redox potentials decrease in order of the expected increase in hydrogen bonding to the ferrocenyl amides from the 0% control to the 100% control. Future work will investigate the use of alternative spectroscopic methods to assay diphenylacetylene switching under redox-mediated conditions, including the exploration of IR marker bands and colorimetric methods.
